# The *TP63* Gene Polymorphism *rs17506395* is Associated with Early Breast Cancer in Cameroon

**DOI:** 10.31557/APJCP.2020.21.8.2199

**Published:** 2020-08

**Authors:** Arnol T Z Tiofack, Gustave Simo, Elvis A Ofon, Ester Dina bell, Chancelin M Kamla, Sidonie N Ananga, Tchamfong Roger, Theophile N Nana, Charlotte T Nguefack, Adamou Fewou, Samuel Takongmo, Smiths Lueong

**Affiliations:** 1 *Molecular Parasitology & Entomology Unit, Department of Biochemistry, Faculty of Science, University of Dschang, Dschang, Cameroun. *; 2 *Medical Oncology, Direction of the Bonassama District Hospital, Douala, Cameroon. *; 3 *Faculty of Medicine and Pharmaceutical Science, University of Douala, Douala, Cameroon. *; 4 *Service of AnatomocytoPathotogy, General Hospital of Douala, Douala, Cameroon. *; 5 *St. Joseph Clinic Cancer Center, Yaounde, Cameroon. *; 6 *Service of Obstetrics and Gynecology, General Hospital of Douala and Faculty of Medicine, University of Buea. *; 7 *Faculty of Medicine and Biomedical Sciences, University of Yaounde, Yaounde, Cameroon. *; 8 *Medico-surgical center of Yaounde-Nsimeyong Hospital, Yaounde, Cameroon. *; 9 *German Cancer Research Center, Essen, Germany. *

**Keywords:** Breast cancer, early onset, TP63, single nucleotide polymorphism, rs17506395, female, Cameroon

## Abstract

**Background:**

Breast cancer (BC) is a leading female cancer worldwide and cause of cancer-related death, especially in developing countries. Genetic predispositions to BC development in African population is poorly studied, and meanwhile the *SNP rs17506395* in *TP63 *gene locus has been associated with the development of breast cancer in Asian women, no investigation has been undertaken within African population. We investigated the impact of this polymorphism in a representative African population.

**Methods:**

We undertook a case-control study including 335 women, of which 111 were breast cancer patients and 224 controls. Using blood-derived germline DNA, PCR-RFLP was used to investigate the polymorphism of *TP63 *gene at *rs17506395* locus. Unconditional logistic regression was used to study the association between the* TP63* gene polymorphism and risk of BC development. After stratification into different age and ethno-linguistic groups as well as menopausal status, the Cochran-Mantel-Haenszel test was used to measure significance of the associations.

**Results:**

Comparing cases with controls, no significant associations between genotype and disease development was observed. Similarly, when cases were stratified according to menopausal status and ethno-linguistic groups, no significant association was observed between genotype and disease development. However, in women of 40 years and below, TT and TG genotypes were associated with breast cancer development. The minor G allele seems to protective against early breast cancer onset OR of 0.5 (95%CI = 0.26-0.94, p = 0.03).

**Conclusion:**

Our data revealed an association between rs15706395 and the risk of early breast cancer onset. The GG genotype seems to reduce the risk of early breast cancer. Larger studies are needed to confirm the potential of this SNP as biomarker for breast cancer prognostic.

## Introduction

Breast cancer (BC) is one of the most common invasive malignancies among women worldwide and is the leading cause of cancer-related-deaths among women (Bray et al., 2018). It constitutes about 25% of cancers diagnosed in women (Bray et al., 2018; Ferlay et al., 2015), and its annual incidence has increased from approximately 1.67 million in 2012 to 2.1 million new cases in 2018 (Bray et al., 2018; Ferlay et al., 2015). In 2012, nearly 60% of BC-related deaths were reported in low-income countries (Ferlay et al., 2015). According to the International Agency for Cancer Research (IARC), breast cancer incidence range from 28 per 100, 000 women in central Africa to more than 37 per 100, 000 women in Western Africa (Bray et al., 2018). Although BC is relatively less prevalent in Sub-Saharan Africa (SSA), survival is also generally poor in this region with high mortality recorded in many settings (Bray et al., 2018; Jedy-Agba et al., 2017).

Currently, African women under the age of 40 years with poor prognostic features are increasingly being diagnosed than older women in sub-Saharan Africa (Dia et al., 2017; Vanderpuye et al., 2017). The age at first diagnosis lies between 20 and 50 years in sub-Saharan Africa (Atangana et al., 2017; Kemfang et al., 2015; Essiben et al., 2016; Sando et al., 2014; Yomi et al., 2011; Ouedraogo et al., 2018) compared with 60 to 70 years in most western societies (Abdulrahman and Rahman, 2012; Ferlay et al., 2013; Karim et al., 2015). In Sub-Saharan Africa, most patients die within one year from diagnosis as a result of disease complications and aggressiveness (Jedy-Agba et al., 2016; Ly et al., 2012). Although the clinic-pathological underpinnings of disease outcomes is still poorly understood, several factors including the poor health infrastructures, the lack of screening programs in some African countries, the late diagnosis, lack of patient support programs and poor clinical among others, may explain the differences observed in breast cancer outcomes in various populations (Jill et al., 2013).

Current evidence suggests that early BC onset in patients below 40 years is clinically and etiologically distinct from breast cancer in older women (Anders et al., 2009; Bleyer et al., 2008; Karihtala et al., 2010). For instance, early onset of BC has specific characteristics such as worse prognosis, high aggressiveness, the higher proportions of high-grade and later-stage tumors, and lower expression of hormone receptor genes (Bleyer et al., 2008; Gnerlich et al., 2009; Jill et al., 2013). Additionally, the “triple-negative” phenotype (ER−, PR−, HER2−) of breast cancer, which seems to be more prevalent in young indigenous African and African-American women is very aggressive, lethal and recurrent (Anders et al., 2009; Eng et al., 2014; Galukande et al., 2014, Sawe et al., 2016). Although the role of accumulated driver and passenger mutations is undisputable, the importance of germline genetic variants in the development of BC is becoming an area of great interest (Mavaddat et al., 2010). Identification of genotypes associated with early disease onset holds the promise of well-tailored surveillance schemes. 

Genetic variants in the TP63 gene locus has been reported to be associated with BC development in Asian populations (Feng et al., 2011; Guan et al., 2012; Zhang et al., 2014). The *TP63* gene is located on chromosome 3q27 and encode for the protein p63 that plays a crucial role in the maintenance of a stem cell population of several epithelial tissues. Although p63 is necessary for the normal development of epithelial organs including mammary glands (Barbareschi et al., 2001), its tissue expression patterns have been shown to be associated with syncytial growth pattern in triple negative breast cancer (Thike et al., 2010). Moreover, a positive association has been reported between the percentages of *p63*-positive cells with a marked nuclear pleomorphism (Thike et al., 2010). At the genetic level, a functional *SNP rs17506395 *(T>G) located in the *TP63* gene, which was initially associated with fertility (Feng et al., 2011) has recently been associated with several cancers including breast cancer in Asian populations (Feng et al., 2011; Guan et al., 2012; Zhang et al., 2014). The TT genotype is associated with poor survival, suggesting that this genotype could be a biomarker of adverse prognosis in breast cancer (Zhang et al., 2014). Despite the aforementioned associations between p63 expression and/or *TP63 *gene polymorphism with BC development and patient outcome, very little, if all any study has addressed this crucial factors on BC development or patient outcome in African women. With this in mind, and mindful of the socio-economic and clinical impacts of BC in developing countries, this study was designed to understand the impact of *TP63* gene polymorphism on the incidence and/or development of BC within an African context.

## Materials and Methods

*Ethical approval and consent to participate*

This study was approved by the Ethics Review and Consultancy Committee (ERCC) of the Cameroon Bioethics Initiative (CAMBIN) under the reference number CBI/395/ERCC/CAMBIN and Protocol number 1086. All participants voluntarily signed written informed consent forms prior to their inclusion into the study and the analysis of participant data was fully anonymized. 

*Study population*

The Cameroonian population is made up of more than 250 ethno-linguistic subgroups from three major ethno-linguistic groups: Bantu (e.g.: Bulu, Bassa, Bakundu, Maka, Douala), Semi Bantu (e.g.: Bamileke, Gbaya, Bamoun, Tikar) and Sudano-Sao (e.g.: Fulbe, Mafa, Toupouri, ShoaArabs, Moundang, Massa, Mousgoum) (Ofon et al., 2017). These different ethno-linguistic groups are spread over several African countries and thus provide an excellent opportunity of studying some global aspects of human genetics research in sub-Saharan Africa (Wonkam et al., 2011). 

For this study, a total of 335 women including 111 patients and 224 controls were prospectively recruited between October 2015 and December 2016 at the oncology and radiotherapy unit of the Douala General Hospital and the St. Joseph clinic cancer center of Yaoundé. All patients were histologically confirmed with invasive breast cancer, but without other clinically detectable neoplasm (case group). Controls were randomly recruited women who had no form of cancer as determined from their medical histories and general physical examination. Women who were below 21 years old and/or not Cameroonian and/or had another type of cancer and/or refused to participate in the study were excluded. Participant information was recorded by means of a structured questionnaire that was provided to the participants after consent was given. Clinical and pathological data for all cases was obtained from the physician and/or collected from hospital records. 

*Blood sampling and DNA Extraction*

About 5 ml of blood sample was taken by vein-puncture into EDTA coated tubes. After centrifugation of each tube at 3000xg for 5 minutes, the buffy coat was collected. From each buffy coat, DNA was extracted using phenol–chloroform-isoamylic alcohol (25:24:1) and then, precipitated with isopropanol. DNA pellets were re-suspended in 50µl of sterile ultrapure water and stored at -20°C until use.

*Genotyping of TP63 gene at rs17506395*

Genotyping was carried out by the Polymerase Chain Reaction-Restriction Fragment Length Polymorphism (PCR-RFLP) where the *TP63* gene was amplified and thereafter, the PCR products were digested with restriction enzyme before their resolution on a 2% agarose gel. 

*Amplification of TP63 gene*

For the amplification of *TP63* gene, the primer pair Rb395F (5-ACAGATAAATTGGTGGAGAGAGAT-3) and Rb395R (5-CACTGTTTGGACCCTGGAA-3) flanking the region containing the SNP rs17506395 were designed using Primer-BLAST Software (Ye et al., 2012). The PCR reactions were carried out in a DNA thermal cycler (Prime, United Kingdom). Each amplification reaction was performed in total volume of 25µL containing 1X PCR buffer (Tris·Cl, KCl, (NH4)2SO4, 1.5 mM MgCl2), 1X Q-Solution (Cat No/ID: 203203 Qiagen, Germany), 20 pmol/L of each primers, 200 nmol/L of each dNTP, 1.5 mM MgCl2, 0.25 U Hot star Taq DNA polymerase (Qiagen), and 5µL of genomic DNA diluted 10 fold. The amplification reactions included a denaturation step at 95°C for 15 min followed by 40 amplification cycles comprising a denaturation step at 95°C for 30s, an annealing step at 58°C for 45s and an extension step at 72°C for 1min. This was followed by a final extension step at 72°C for 10 min. For every PCR reaction, a negative control was included where the nuclease free water was used instead of the DNA. The amplified products were resolved by electrophoresis at 135 volts for 30 min on 2% agarose gel containing ethidium bromide. The gels were visualized under ultraviolet light and then photographed using a gel documentation system (UVIsave HD5, Unitec Cambridge). Samples with a successful amplification of the target region (450bp fragment) were selected and subjected to digestion with restriction enzyme. 

*Digestion of PCR products*

For this digestion, 10µL of PCR product were mixed with 10 units (1µL) of MboII restriction enzyme (Cat #: R0148L; Lot number: 0911511, New England Biolabs, Inc.). The digestion was performed for 18 hours at 37°C. The digested products were subsequently resolved on a 2% agarose gel containing ethidium bromide. Samples presenting two DNA fragments of 235 and 215bp were considered as homozygote wild type with genotype TT while those showing a single DNA fragment of 450bp were considered as homozygous mutant with genotype GG. A profile showing three DNA fragments of 235, 215, and 450bp were considered as heterozygote with genotype TG.

*Power calculation*

For this study, we assumed an additive genetic model where two risk alleles of an SNP (homozygous) have twice the effect of one risk allele (heterozygous) (Menashe et al., 2008). Power calculations were undertaken using the PGA modeller package in MATLAB (Menashe et al., 2008). For this Software, the power was calculated by considering an odd ratio (OR) or relative risk (RR) ≥ 2 for locus with disease allele frequency of 0.1612-0.205 with one locus genotyped. Other factors taken into consideration include the disease prevalence estimated at 25% according to GlOBOCAN 2012 (Ferlay et al., 2015), a type 1 error of 5% risk, a complete linkage parameter (r^2^) for linkage disequilibrium (LD) of 1.0 and the sampling size. This later was estimated as described by Kasiulevicius et al., (2006) using the independent case-control sampling size formula. For this estimation, we assumed an expected exposure proportions in control of 20%, a disease prevalence of 25% (Ferlay et al., 2015) and a case-control ratio of 1:2. With the independent case-control sampling size formula, the sampling size to detect a real odds ratio or case exposure rate with power and two-sided type I error of 5% risk was 195 including 65 breast cancer patients and 130 controls.

*Association studies*

Before the association studies, the Hardy-Weinberg equilibrium test (HWE) was undertaken not only on the entire population, but also on different subpopulations stratified by ages, menopausal status and ethno-linguistic subgroups. 

To test if there was any association between polymorphism at *SNP rs17506395* of *TP63* gene and the risk of breast cancer development, the association analysis were firstly performed on the entire population made up of 111 patients and 224 controls. For these association studies, the unconditional logistic regression models were used to estimate odds ratio (OR) at 95% confidence intervals (CI) in the entire population without any stratification as well as in each stratified population.When the absence of one genotype or allele (represented by zero) has led to problems with the computation of the odds ratio (standard error), 0.5 was added to all cells (values involved in formula) as supposed by Deeks and Higgins (2010) (Pagano and Gauvreau, 2000; Deeks and Higgins, 2010). Pearson chi-square (*χ*^2^) tests and the Fisher’s exact test were used to compare categorical variables between the participants while the student t-test was used to compare the mean values for continuous variables between groups. The test was considered significant for a P value below 0.05.

To see if the heterogeneous nature of our study population, made up of women having different menopausal status or belonging to different age groups or various ethno-linguistic subgroups, could have an effect on the results of association studies, additional analyses were undertaken on different subpopulations. Each subpopulation was considered here as all women belonging to the same ethno-linguistic group or the same age group or having the same menopausal status. These analyses were performed when the participants were subdivided according to their menopausal status, their ethno-linguistic groups (Bantu, semi-Bantu and Sudano-sao) and their age group (women ≤ 40 years old and those above 40). For these stratified population, the Cochran-Mantel-Haenszel (CMH) test implemented in PLINKv1.9 package (Purcell et al., 2007) was performed with the allelic frequencies because this test can only be done with binary vars. The CMH test enabled not only to estimate the odds ratio and 95% confidence interval across the stratified population, but also to test the associations between alleles and the probability to develop a breast cancer in each stratum. Moreover, the CMH2 test, also implemented in PLINKv1.9 package, was used to determine if there were significant differences between the allele frequencies of different subpopulations. The genotype-based Cochran-Armitage trend test with high power and two degrees of freedom (in the 2 x 3 crosstab) was performed to test the association between rs17506395 and the susceptibility to breast cancer. The statistical analyses were conducted using SPSS Software 22.0 (SPSS Inc., Chicago, Illinois, USA) and PLINK v1.9 package. The chi-squared test was used to compare the allelic and genotypic frequencies between breast cancer patients and controls.

## Results

*Socio-demographic and clinical characteristics of the study population*

In our study, 335 Cameroonian women were recruited: 111 breast cancer patients who were histologically confirmed with infiltrating ductal carcinomas and 224 controls (Table 1). Amongst the 335 participants, 74 (22.09%) were Bantu, 254 (75.82%) semi Bantu and 7 (2.09%) Sudano-Sao. The age of breast cancer patients at diagnosis ranged from 24 to 72 years with a mean of 41.64 (SD =12.31) years while those of the controls were range from 25 to 78 years with a mean of 39.55 (SD = 10.63) years. No significant difference was observed between mean age of patients and controls (p-value= 0.11). Considering the ethno-linguistic groups, the menopausal status as well as familial BC, significant differences (p<0.001) were observed between patients and controls (Table 1). 

From the 111 breast cancer patients, 77 (69.37 %) were premenopausal and 53 (47.7%) were below 40 years. About 91.89% (102/111) all patients were either at stage III or IV while only 8.11% (9/111) were either at stage I or II. Seventy one (63.96%) patients had lymph node involvement. Grouping the patients according to lymph node status and tumor size, significant different (p <0.0001) were observed (Table 1). About 36.94% (41/111) of patients were found with breast cancer at the metastatic stage (Table 1). 

Of the 224 controls, 195 (87%) were premenopausal and 12.95% postmenopausal women. About 58.04 % (130/224) of these controls were 40 years or less (Table 1). 

With a LD r2 of 1.0, a disease prevalence of 25%, the disease allele frequencies range from 0.1612-0.205 for one locus genotyped, and a sampling size of 335 individuals including 111 breast cancer patients and 224 controls, the power of this study was estimated at 95%.

*Genotyping results*

The polymorphism at *SNP rs17506395 *locus of* TP63 *was investigated on 111 breast cancer patients and 224 controls belonging to three ethno-linguistic groups. The primers designed in this study successfully amplified all the 335 samples. 

*Allelic frequencies*

For the entire population, the allelic frequency was 83.9% (562/670) for T allele and 16.1% (108/670) for G. In breast cancer patients, the allelic frequencies for T and G alleles were 86.9% (193/222) and 13.1% (29/222) respectively. In the controls, the allelic frequency was 82.4% (369/448) and 17.6% (79/448) for T and G allele, respectively (Table 2). Between patients and controls, no significant difference was observed (p = 0.13) in the distribution of the different alleles.

In patients of 40 years or less, the allelic frequencies were respectively 86.79% (92/106) and 13.21% (14/106) for T and G alleles. In controls of the same age group, the frequencies of T and G alleles were respectively 76.54% (199/260) and 23.46 (61/260). Between controls and patients of 40 years or less, a significant difference (p = 0.03; OR = 0.5, 95%CI = 0.26-0.94) was observed in allelic distribution. These frequencies were 87.07% (101/116) and 12.93% (15/116) respectively for T and G alleles in patients above 40 years. For controls of the same group of ages, the frequency of the T allele was 90.43% (170/188) while that of G was 9.57% (18/188). In women above 40 years, no significant difference (p = 0. 313; OR = 1.473, 95%CI = 0.6747-3.215) was observed.

In premenopausal patients, the allelic frequency was 87.66% (135/154) for T allele and 12.34% (19/154) for G. In controls with the same status, the allelic frequencies for T and G alleles were respectively 81.03% (316/390) and 18.97% (74/390). For postmenopausal patients, the T allele has an allelic frequency of 85.29% (58/68) while that of G allele was 14.71% (10/68) (Table 2). In controls, the allelic frequency was 91.38% (53/58) for T allele and 8.62% (5/58) for G (Table 2). No significant difference was observed in the allelic frequencies between patients and controls having pre and postmenopausal status. 

The distribution of T and G alleles among the different ethno-linguistic groups is summarized in Table 2. In patients, the allelic frequencies for the T allele were 83.33% (55/66) in the Bantu ethno-linguistic group, 88.19% (127/144) in the semi-Bantu and 91.67% (11/12) in the Sudano-sao group. In controls, these allelic frequencies were 79.27% (65/82) in Bantu ethno-linguistic group, 82.97% (302/364) in the semi-Bantu and 100% (2/2) in the Sudano-sao group. For the G allele, the allelic frequencies in patients were 16.67% (11/66) in the Bantu, 11.81% (17/144) in the semi-Bantu and 8.33% (1/12) in the Sudano-sao. For the same ethno-linguistic groups, similar trends were observed for the allelic frequencies in controls: 20.73% (17/82) for the allelic frequency in the Bantu ethno-linguistic group, 17.03% (62/364) for the-Bantu and none for the Sudano-sao (Table 2). However, not all of these distributions are statistically significant (Table 2).

*Genotypic frequencies*

For the entire population, the genotypic frequencies were 24.5% (92/335) for TG genotype (heterozygote), 2.4% (8/335) for GG genotype (homozygote mutant) and 67.2% (235/335) for TT genotype (homozygote wild type) (Table 3). In patients, 73.90% (82/111) were of the TT genotype and 26.10% (29/111) TG genotype (Table 3). In the controls, 68.30% (153/224) and 3.60% (8/224) of women were homozygote wild type and mutant respectively while 28.10% (63/224) were heterozygotes (Table 3). In the entire population, no significant difference was observed (p = 0.13 and 0.56 for GG and TG respectively) in the genotypic distribution between patients and controls (Table 3).

The GG genotype was present only in controls of 40 years or less (Table 3). About 73.58% (39/53) of patients of 40 years or less were homozygote wild type while 26.42% (14/53) were heterozygote. In controls of the same age, 34.62% (45/130) were heterozygote while 59.23% (77/130) and 6.15% (8/130) were homozygote wild type and mutant respectively. Between controls and patients of 40 years or less, a significant difference (p = 0.03) was observed in their genotypic frequencies (Table 3). In patients above 40 years, the genotypic frequencies were 74.14% (43/58) for the homozygote wild type and 25.86% (15/58) for the heterozygote. In controls of the same age, 80.85% (76/94) and 19.15% (18/94) were respectively homozygote wild type and heterozygote. No participant above 40 years (either patients or controls) carried the homozygote mutant genotype (Table 3). No significant difference (p = 0.33) was found for the genotypic frequencies between patients and controls who were above 40 years (Table 3). 

From the 77 premenopausal patients, the genotypic frequencies were 75.32% (58/77) for TT genotype and 24.68% (19/77) for TG genotype. None of these women was found with GG genotype. In 195 controls of the same menopausal status, the genotypic frequencies were 66.15% (129/195) for TT genotype, 29.74% (58/195) for TG genotype and 4.10% (8/195) for GG genotype. In postmenopausal patients, the genotypic frequencies were 70.59% (24/34) for the TT genotype and 29.41% (10/34) for TG genotype. For controls having the same menopausal status, the genotypic frequencies were 82.76% (24/29) for TT genotype and 17.24% (5/29) for TG genotype (Table 3). No postmenopausal woman was found with GG genotype either in patient or in controls (Table 3). Comparing the genotypic frequencies between patients and controls in pre and postmenopausal women, no significant difference was observed (Table 3).

The GG genotype was only found in controls of the Bantu and semi-Bantu ethno-linguistic groups with respective genotypic frequencies of 7.32% (3/41) and 2.75% (5/182). In contrast, other genotypes were present in all ethno-linguistic groups (Table 3). In fact, for the patients of the semi-bantu group, the genotypic frequencies were 76.39% (55/72) and 23.61% (17/72) for TT and TG genotypes respectively. In controls of this group, we found 68.68% (125/182) and 28.57% (52/182) for TT and TG genotypes respectively. 

Among the Bantu group, the TT and TG genotypes have respective genotypic frequencies of 66.67% (22/33) and 33.33% (11/33) for patients; and 65.85% (27/41) and 26.83% (11/41) for controls. The genotypic frequencies for TT and TG genotypes were respectively 83.33% (5/6) and 16.67 (1/6) for patients in the Sudano-sao group. The only one control of this group has TT genotype. Whatever, the ethno-linguistic group, no significant difference was observed in the genotypic frequencies between patients and controls.. 

*Association between polymorphism at SNP rs17506395 and the risk of developing breast cancer in the whole population*

The overall population was in Hardy–Weinberg equilibrium (p =1). The Cochran-Armitage trend test performed using the genotypic frequencies of the overall study population revealed no significant association (p = 0.1269) between the polymorphism at *SNP rs17506395 *and the risk of developing breast cancer in our study population (Table 4). These results are in agreement with those of the allelic frequencies reporting no significant difference between patients and controls (p = 0.1287, OR = 0.7, 95% CI = 0.44-1.11) (Table 3). Whatever the genotype considered, no significant difference (genotype TG: p = 0.562, OR = 0.86, 95% CI = 0.510-1.44; genotype GG: p = 0.130, OR = 0.11, 95% CI = 0.006-1.920) was found between patients and controls (Table 4).

To see if these results could be bias by the heterogeneity of the study population, additional analyses were carried out on the populations stratified on the basic of their menopausal status, ethno-linguistic groups as well as age groups. 

*Association between polymorphism at SNP rs17506395 and the risk of developing breast cancer in women of different Subpopulations*

Data from the Cochran-Mantel-Haenszel (CMH) and CMH2 tests reported in Table 4 includes all 111 patients and 222 controls. The null hypothesis of the CMH test is that the allele frequencies are the same in cases and controls and do not differ between populations. Whatever the subpopulation considered in the association studies performed with the CMH test, the minor allele G of rs17506395 was not associated (p > 0.1) with breast cancer development (Table 4). However, with the CMH2 test for which the null hypothesis is described by the same allele frequencies in each population, a significant difference (P = 0.00116, *X*^2^ = 10.55) was observed in allele frequencies between the age groups. The odds ratio at 95% confidence intervals estimated using the unconditional logistic regression models are reported in Table 2. No significant association was observed for the subpopulations subdivided on the basis of their menopausal status and Ethno-linguistic groups (Table 4).

*Association between polymorphism at SNP rs17506395 of TP63 and the risk of developing breast cancer in women of different age groups*

The Cochran-Armitage trend test found an association (Cochran-Armitage P value= 0.03) between genotype combinations ([TT + TG] versus [GG]) and the risk of breast cancer development in women of 40 years and less (Table 3). The GG genotype appears to reduce the risk of breast cancer development among women 40 years or less (Table 3). The unconditional logistic regression analysis revealed that women of 40 years or less and who carry the G allele had a significantly decreased risk (p = 0.0311, OR = 0.5, 95% CI = 0.26-0.94) of developing breast cancer compared to those carrying T allele (Table 2). This result indicates that the G allele has a protective effect on the development of breast cancer in this group of women. For women above 40 years, no association (Cochran-Armitage P value = 0.33) was observed between the number of variant alleles and the risk of developing breast cancer (Table 3).

*Association between polymorphism at rs17506395 and the risk of developing breast cancer in women of different menopausal status*

For this association study, the participants were subdivided into 272 premenopausal women (77 breast cancer patients and 195 controls) and 63 postmenopausal women (34 patients and 29 controls). Using the unconditional logistic regression analysis, no association (p= 0.067, OR= 0.5996? 95%CI 0.3471-1.036) was observed for the allelic frequencies in premenopausal women (Table 3). The Cochran-Armitage trend test and the unconditional logistic regression models revealed also no association between the number of variant alleles and the risk of developing breast cancer among women of this subpopulation (Tables 3 and 4). In the postmenopausal women, no association was found when different tests were applied on the allelic frequencies (p= 0.263, OR=2, 95CI%= 0.5943-6.73) or the genotypic frequencies (for TG, p= 0.262, OR= 2, 95CI%=0.594-6.730 and for GG, p= 1, OR= 1, 95%CI= 0.019-52.440). These results were confirmed by the Cochran-Armitage trend test (Table 3).

*Association between polymorphism at rs17506395 and the risk of developing breast cancer in different to ethno-linguistic groups*

Between patients and controls, and within the different ethno-linguistic groups, no significant difference was found in the allelic frequencies when the unconditional logistic regression model was used (Table 2). The Cochran-Armitage trend test performed between patients and controls belonging to different ethno-linguistic groups also revealed no association (Cochran-Armitage p >0.1) between the number of variant alleles and the risk of developing breast cancer in these subpopulations (Table 3).

Additional association studies with the tests used above revealed no significant association between polymorphism at* SNP rs17506395* of *TP63* gene and the risk of developing breast cancer in women presenting different clinico-pathological features of the disease (Table 5).

**Table 1:** Socio-Demographic and Clinical Characteristics of the Study Population

Clinical	Cases, n=111 (%)	Controls, n=224 (%)	*P*-value
Age group			0.11
> 40	58 (52.3)	94 (41.96)	
≤ 40	53 (47.75)	130 (58.04)	
Age (means±SD)	41.64±12.31	39.55±10.63	
Ethno-linguistic group			<0.001
Bantu	33 (29.73)	41 (18.30)	
Semi Bantu	72 (64.86)	182 (81.25)	
Sudano-sao	6 (5.41)	1(0.45)	
Menopause status			<0.001
postmenopausal	34 (30.63)	29 (12.95)	
Premenopausal	77 (69.37)	195 (87.05)	
Familial breast cancer			<0.001
Yes	29 (26.13)	16 (7.14)	
No	82 (73.87)	208 (92.86)	
Histological grade			
I, II	9 (8.11)	-	
III, IV	102 (91.89)	-	
* P*-value	<0.0001		
Lymph node			
Yes	71 (63.96)	-	
No	40 (36.04)	-	
* P*-value	<0.0001		
Metastasis			
Yes	41 (36.94)	-	
No	70 (63.06)	-	
* P*-value	<0.0001		

**Table 2:** Allelic Frequencies in Breast Cancer Patients and Controls According to Subpopulations

Subpopulation	Alleles	Patients (AF)	Controls (AF)	OR	95%CI	*P*-value
Whole population	T	193 (86.94)	369 (82.40)	-	-	0.129
	G	29 (13.10)	79 (17.60)	0.70	0.44-1.11	
≤40 years	T	92 (86.79)	199 (76.54)	-	-	0.0311
	G	14 (13.21)	61 (23.46)	0.50	0.26-0.94	
>40 years	T	101 (87.07)	170 (90.43)	-	-	0.331
	G	15 (12.93)	18 (9.57)	1.473	0.6747-3.215	
Premenopausal	T	135 (87.66)	316 (81.03)	-	-	0.067
	G	19 (12.34)	74 (18.97)	0.5996	0.3471-1.036	
Postmenopausal	T	58 (85.29)	53 (91.38)	-	-	0.263
	G	10 (14.71)	5 (8.62)	2	0.5943-6.73	
Bantu	T	55 (83.33)	65 (79.27)	-	-	0.538
	G	11 (16.67)	17 (20.73)	0.7703	0.3359-1.766	
Semi-Bantu	T	127 (88.19)	302 (82.97)	-	-	0.139
	G	17 (11.81)	62 (17.03)	0.6419	0.357-1.154	
Sudano-sao	T	11 (91.67)	2 (100)	-	-	0.81
	G	1 (8.33)	0 (0.00)	0.6522	0.020-21.183	

AF, Genotypic frequency; OR, odd ratio; CI, confidence interval

**Table 3:** Genotypic Frequencies in Breast Cancer Patients and Controls According to Subpopulations

Subpopulation	Genotypes	Patients (GF)	Controls (GF)	OR	95%CI	P value	Cochran-Armitage p-value
Whole population	TT	82 (73.90)	153 (68.30)	-	-	-	0.1269
	TG	29 (26.10)	63 (28.10)	0.86	0.510-1.44	0.562	
	GG	0 (0.00)	8 (3.60)	0.11	0.006-1.920	0.13	
≤40 years	TT	39 (73.58)	77 (59.23)	-	-	-	0.03
	TG	14 (26.42)	45 (34.62)	0.61	0.30-1.25	0.1802	
	GG	0 (0.00)	8 (6.15)	0.11	0.007-2.052	0.141	
>40 years	TT	43 (74.14)	76 (80.85)	-	-		0.33
	TG	15 (25.86)	18 (19.15)	1.5	0.674-3.21	0.331	
	GG	0 (0.00)	0 (0.00)	1.76	0.034-90.210	0.78	
Premenopausal	TT	58 (75.32)	129 (66.15)	-	-	-	0.06
	TG	19 (24.68)	58 (29.74)	0.73	0.398-1.332	0.304	
	GG	0 (0.00)	8 (4.10)	0.13	0.074-2.29	0.164	
Postmenopausal	TT	24 (70.59)	24 (82.76)	-	-	-	0.26
	TG	10 (29.41)	5 (17.24)	2	0.594-6.730	0.262	
	GG	0 (0.00)	0 (0.00)	1	0.019-52.440	1	
Bantu	TT	22 (66.67)	27 (65.85)	-	-	-	0.54
	TG	11 (33.33)	11 (26.83)	0.44	0.179-1.06	0.07	
	GG	0 (0.00)	3 (7.32)	0.17	0.0086-3.560	0.25	
Semi-Bantu	TT	55 (76.39)	125 (68.68)	-	-	-	0.14
	TG	17 (23.61)	52 (28.57)	0.74	0.395-1.399	0.3575	
	GG	0 (0.00)	5 (2.75)	0.2	0.011-3.782	0.287	
Sudano-sao	TT	5 (83.33)	1 (100)	-	-	-	0.66
	TG	1 (16.67)	0 (0.00)	0.818	0.021-32.270	0.915	
	GG	0 (0.00)	0 (0.00)	0.3	0.0036-20.420	0.555	

GF, Genotypic frequency; OR, odd ratio; CI, confidence interval

**Table 4:** Cochran-Mantel-Haentel (CMH) Association Analysis Results within each Subpopulation

Strata	alleles	MAF	*P*-value	CHISQ	^a^P-value	^a^CHISQ	OR (95%CI)
Age group	*T	0.1612	0.2261	1.465	0.00116	10.55	0.7507 (0.471-1.194)
	**G						
Menopausal status	*T	0.1612	0.2133	1.549	0.249	1.32	0.74 (0.459-1.185)
	**G						
Ethno-linguistic group	*T	0.1612	0.1256	2.346	0.371	1.98	0.6912 (0.4312-1.108)
	**G						

*P-*value, Nominal CMH P asymptotic probability value; **, minor allele; *major allele, CHISQ: Chi-square probability value; OR: odds ratio; MAF, Minor allele frequency; ^a^, Cochran-Mantel-Haenszel for homogeneity of association across clusters (using the cmh2 test in plink

**Table 5:** Relationship between rs17506395 and Known Clinicopathological Variables

Clinicopathological variables	Number (n=111)	Genotype (%)		*P*-value
		TG	TT	
Age (years)				0.947
≤40	53	14 (26.42)	39 (73.58)	
>40	58	15 (25.86)	43 (74.14)	
Site of Breast				0.882
Left	53	15 (28.30)	38 (71.70)	
Right	54	13 (24.07)	41 (75.93)	
Bilateral	4	1 (25)	3 (75)	
Tumor stage				0.192
I, II	9	4 (44.44)	5 (55.60)	
III, IV	102	25 (24.51)	77 (75.49)	
Lymph node				0.27
Yes	71	21 (29.58)	50 (71.42)	
No	40	8 (20)	32 (80)	
Metastasis				0.306
Yes	41	13 (31.71)	28 (68.29)	
No	70	16 (22.86)	54 (77.14)	

## Discussion

The evaluation of genetic variants associated with the risk of BC development has led to a better understanding of the relationship between genomics and cancer development. Such relationships have been fundamentally based on SNPs, which generally carry the most common pattern of genetic variation in the human genome (Chang-Sheng et al., 2018). Zhang et al., (2014) have shown an association between polymorphism at *SNP rs17506395* of the *TP63* gene and the breast cancer in Asian population. Although the definition of young women with breast cancer is still controversial with different authors defining different ages as young and varying from 30 to 45 years (Bollet et al., 2007; Kruger and Apffelstaedt, 2009), all women of 40 years and below were considered in our study as young, otherwise they were of old age. Our classification is in agreement with age stratifications that have recently been used in several studies (Bharat et al.,, 2009; Dia et al., 2017; Johnson et al., 2015; Karihtala et al., 2010; Lian et al., 2017).

Our results showing that the T allele seems to be widely spread in Cameroonian Women are in agreement with those obtained from the 1000 Genomes project where the T allele represents 89% against 11% for G allele (Hunt et al., 2018). However, the present study shows a significant difference (p = 0.03) in the allelic frequencies of T allele between patients and controls for women of age 40 years and or less. On the other hand, no significant difference was observed between controls and patients of above 40 years as well as in the entire population or participants grouped according to their ethno-linguistic groups or menopausal status. The presence of T allele seems to have an impact on breast cancer outcome in women of 40 years and less. This hypothesis is in line with observation of Zhang et al. (2014) reporting high frequency of T allele in breast cancer patients. In Asian women for instance, Zhang et al., (2014) observed 70.33% of T allele in controls against 78.69% in patients. In our study, such difference was observed only in participants of 40 years and less (86.79% for patients versus 76.54% for controls). From the differences in the allelic frequencies, it was inferred that the single present of T allele seems sufficient to increase the risk of developing breast cancer in Asian women. The presence of T allele seems therefore to be associated with the risk of developing breast cancer in women of 40 years and less. Although the mechanism associating the presence of T allele at *SNP rs17506395 *with breast cancer is not yet understood, this allele seems to be able to alter the regulator power of *TP63* gene, which could contribute to breast cancer development (Zhang et al., 2014). It was suggested that the T allele, which is mostly observed in breast cancer patients, may enhance the expression of *TP63* gene. If we assume that, it is likely that the high frequency of T allele among young patients may promote high expression of TP63, which in tune drive the aggressiveness of basal-like breast cancer. Previous studies highlight that the total percentage of p63-positive cells was related to a marked nuclear pleomorphism and that the intensity of *p63* staining was associated with a syncytial growth pattern in triple negative breast cancer (Thike et al., 2010). Moreover, higher expression of p63 was reported in the cytoplasm cells of basal-like subtype breast cancer compared to non-basal-like of Indonesian women (Kamarlis et al., 2018). Results obtained in young Cameroonian patients highlighting the high allelic frequency for T allele could be linked to aggressiveness of breast cancer in the young black women because breast cancer in women of below 40 years is more aggressive with a worse clinical outcome compared to older group (Anders et al., 2010; Carvalho et al., 2010; Karihtala et al., 2010). The exact mechanism underlying the function of this polymorphism has not been unveiled and functional studies are required in order to understand the biological function related to the polymorphism observed at SNP of *rs17506395* of* TP63* gene.

In our study, the G allele seems to be predominant in the young population because its allelic frequency was 23.46% and 13.21% in controls and patients of 40 years and less against 9.57% and 12.93% in those above 40 years. These results are in agreement with those obtained in Asian women where Zhang et al., (2014) reported 50.4% of young women (below 48 years) carrying G allele against 17.3% in patients above 48 years although the grouping by age was different. In the present study, the allelic frequency for G allele was significantly high in controls than patients of 40 years and less. However, no significant difference was observed for the allelic frequencies between controls and patients when all participants or the ethno-linguistic groups or the menopausal status were taken into consideration. These results suggest the implication of G allele in the breast cancer outcome in participants of 40 years and less. For this allele, an odds ratio of 0.5 and a significant p value of 0.03 suggest that the presence of G allele could reduce the risk of developing breast cancer in the young Cameroonian women. This allele may have a protecting effect against breast cancer in Cameroonian women of 40 years and less; this effect has been reported elsewhere by Zhang et al., (2014).

The Cochran–Armitage test for trend revealed an association between the combination of TT and TG genotypes with the risk of developing breast cancer in of women of 40 years and less. These results are in agreement with those of Zhang et al., (2014). These authors reported the TT genotype to be associated with unfavorable survival of breast cancer; suggesting this genotype as potential biomarker for adverse prognosis of breast cancer in Asian women. According to results of Cochran-Armitage trend test on the group of women of 40 years and less, the GG genotype seems to be associated (p=0.03) with a decreasing risk of developing breast cancer. These results are in agreement with those obtained by Zhang et al., (2014) who found that breast tumors with TT genotype exhibited higher level of *TP63 mRNA *compared with other genotypes in breast cancer tissues, indicating that *rs17506395* may be a functional single nucleotide polymorphism in breast cancer. The presence of TT genotype was statistically correlated with increased risk for breast cancer, compared with genotypes containing the G allele (GG and GT). If the presence of T allele may enhance the expression of the *TP63* gene, such phenomenon may subsequently drive tumorigenesis than the GG and GT genotypes could do (Zhang et al., 2014). Moreover, an increase in the expression level of *TP63* gene is generally observed in less differentiated or more aggressive tumors, which would indicate the correlation between an increase in p63 expression and a poor prognosis (Hsiao et al., 2010). Therefore, the association of this functional variant *rs17506395* of the *TP63* gene in young Cameroonian women would partially justify the aggressiveness of cancer among young black women in sub-Saharan Africa. 

Our study failed to find significant association between polymorphism at *rs15706395* of *TP63* gene and risk of developing breast cancer in different ethno-linguistic groups. These results suggest that the *rs15706395* variant of the* TP63* gene could exert its effect regardless of ethno-linguistic groups. Although the power of our study is 95%, it is important to point out that the low sample size of the sudano-soa ethno-linguistic group might have an impact on the outcome of this study. This low sample size of women belonging to the Sudano-sao group could partially be explained by some cultural behavior and believes. Indeed, women belonging to this ethno-linguistic group are generally reluctant to participate in Breast and cervical cancer screening programs (Banning et al., 2009; Padela et al., 2016). Additional studies need to be conducted in larger sample sizes to validate these findings.

In conclusion, results of this study revealed an association between polymorphism at *SNP rs15706395 *of *TP63* gene and the risk of developing breast cancer among Cameroonian women of 40 years and less. The GG genotype seems to reduce the risk of developing breast cancer in young Cameroonian women. Further studies using larger sample sizes are needed to provide more conclusive evidences. Such studies could enable to confirm the potential of this SNP as biomarker for breast cancer prognostic. 
